# Ultrasound as a Tool to Study Muscle–Tendon Functions during Locomotion: A Systematic Review of Applications

**DOI:** 10.3390/s19194316

**Published:** 2019-10-05

**Authors:** Christoph Leitner, Pascal A. Hager, Harald Penasso, Markus Tilp, Luca Benini, Christian Peham, Christian Baumgartner

**Affiliations:** 1 Institute of Health Care Engineering with European Testing Center of Medical Devices, Graz University of Technology, Stremayrgasse 16/II, 8010 Graz, Austria; 2 Institute of Sport Science, University of Graz, Mozartgasse 14, 8010 Graz, Austria; 3 Integrated Systems Laboratory, ETH Zürich, Gloriastrasse 35, 8092 Zürich, Switzerland; 4 Electrical, Electronic and Information Engineering - DEI, Università di Bologna, Viale del Risorgimento 2, 40136 Bologna, Italy; 5 Department for Companion Animals and Horses, University of Veterinary Medicine Vienna, Veterinärplatz 1, 1210 Wien, Austria

**Keywords:** ultrasound, system design, form factor, range of view, frame rate, in vivo, biomonitoring, human and animal locomotion, muscle, tendon, fascicle, velocity

## Abstract

Movement science investigating muscle and tendon functions during locomotion utilizes commercial ultrasound imagers built for medical applications. These limit biomechanics research due to their form factor, range of view, and spatio-temporal resolution. This review systematically investigates the technical aspects of applying ultrasound as a research tool to investigate human and animal locomotion. It provides an overview on the ultrasound systems used and of their operating parameters. We present measured fascicle velocities and discuss the results with respect to operating frame rates during recording. Furthermore, we derive why muscle and tendon functions should be recorded with a frame rate of at least 150 Hz and a range of view of 250 mm. Moreover, we analyze why and how the development of better ultrasound observation devices at the hierarchical level of muscles and tendons can support biomechanics research. Additionally, we present recent technological advances and their possible application. We provide a list of recommendations for the development of a more advanced ultrasound sensor system class targeting biomechanical applications. Looking to the future, mobile, ultrafast ultrasound hardware technologies create immense opportunities to expand the existing knowledge of human and animal movement.

## 1. Introduction

**Figure 1:**
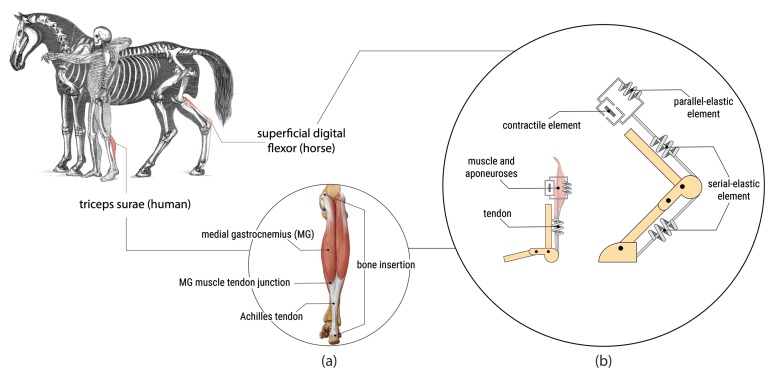
Components and modelling of Hill-type muscle–tendon units (MTUs) in humans and horses: (**a**) shows the human plantarflexor triceps surae (TS) and the components and landmarks of the medial gastrocnemius (MG) MTU; (**b**) shows a simple modeling approach [12] of the human MG MTU and the equine superficial digital flexor (SFDF) MTU [11]. An alignment of elastic springs and contractile elements is used to model the functions of muscles (active) and tendons (passive).

Species living on Earth are exposed to gravity. As a result of the cyclic nature of locomotion and due to gravitational force, muscle–tendon structures (Figure 1) repeatedly function in eccentric (elongation) and concentric (shortening) ways, producing force in both conditions. The combination of eccentric and concentric actions in tissues forms a natural type of muscle and tendon function known as the stretch–shortening cycle (SSC) [1]. Studies from the 1980s and early 1990s conceived the SSC as a muscle function during human walking and running [1,2], without being able to actually observe the structure during movement. Newer studies performed with ultrasound (US) were able to capture the distinct contributions of muscles and tendons within the muscle–tendon unit (MTU), respectively.

The MTU is not one uniform unit with consistent properties, but a mixture of active/passive and contractile/visco-elastic elements with different properties and roles during the SSC. Besides force generation in muscles, elastic energy storage and return is a basic principle in human and animal locomotion [3,4,5]. Studies have shown that the MTU can elongate even if the muscle is isometrically or concentrically contracted [6,7,8,9]. Contractile elements of particular MTUs used for locomotion in horses [10] and to a greater extent in camels [11] are reduced to a rudiment. This leaves the remaining tendon structures running virtually uninterrupted from origin to insertion. Hence, the lengthening and shortening of muscle fascicles contribute little to the total length change of the MTU. In conclusion, the interaction of the whole MTU is responsible for the resultant movement.

Ultrasound found its way into movement science laboratories in the 1990s. As a research tool US has advantages over other tissue imaging techniques. Most significantly, US is a direct, non-invasive, in-vivo method in which test subjects are not exposed to radiation. Hence, even long-duration tests can be repeated frequently [13]. The use of US imagers allows the direct and real-time examination of tissues and their response to mechanical stress and muscle contraction by looking inside the body [14]. During movement, tendons and aponeuroses transmit forces from contracting muscles to the bone and act as biological springs, storing and releasing elastic energy [5,15]. As such, US provides a valuable tool for understanding how muscles and tendons interact or might get injured due to acute or chronic loading [14].

Recently, 2d B-Mode US has also been applied to investigate (biomonitor) muscle and tendon dynamics in complex movement tasks in humans (e.g., walking, running, and jumping). Currently, movement science utilizes clinical US devices for research purposes. US imagers built for medical applications are not entirely adequate for direct in-vivo measurements of muscle and tendon dynamics, as stated in previous reviews [14,15,16,17]. They limit biomechanical research mainly by their form factor, range of view, and spatio-temporal resolution. This restricts the ability to biomonitor the whole muscle–tendon complex and its non-linear behavior. Advances in human and animal locomotion research depend on the development of new and better observation devices and sensors at any hierarchical level of the body [16].

This article investigates US-based biomonitoring (2d B-Mode) of muscle and tendon dynamics during locomotion. We review the state-of-the-art and shortcomings of the current technology. Furthermore, we take a closer look at the system designs and form factors of US platforms and derive why muscle and tendon functions should be recorded with frame rates of at least 150 Hz and a range of view of 250 mm. Moreover, we provide a list of suggestions for future developments of a US system class targeting biomechanics research.

The Materials and Methods of the systematic investigation are presented in Section 2 with supplementary material in Appendix A. Section 3 outlines which US platforms and parameters are currently used for investigating muscle and tendon structures during locomotion. Furthermore, we provide an overview of measured fascicle velocities in lower limbs and present the results in the context of operating frame rates during recording. Section 4 discusses these results and proposes future engineering directions for US systems in movement science research.

## 2. Materials and Methods

**Figure A1:**
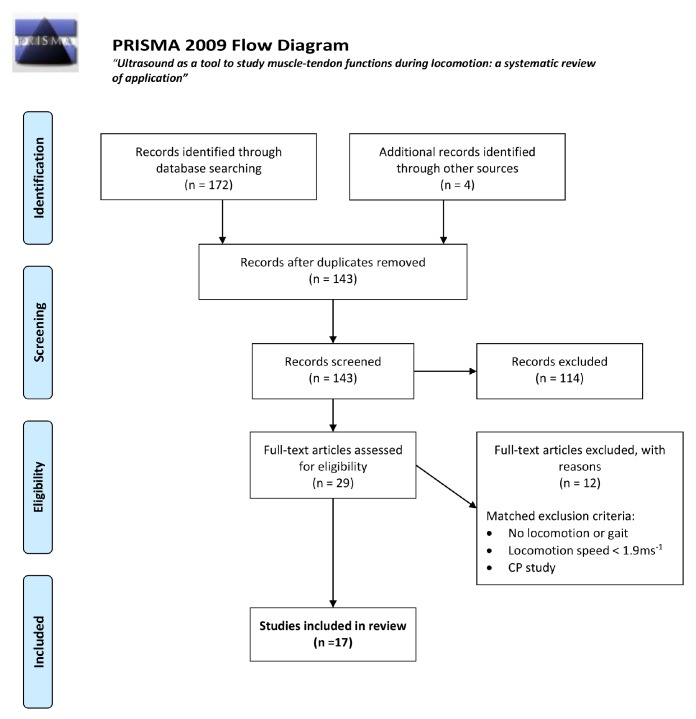
The present review followed the PRISMA Flow Diagram [18].

A systematic approach based on the PRISMA guidelines was used [18]. The database search including SCOPUS, MEDLINE (PubMed), and Google Scholar led to the inclusion of 17 studies (Figure A1). All identified studies (Table 1) investigated muscle and tendon complexes in healthy humans using US during locomotion speeds higher than 1.9 ms${}^{-1}$. Applying the same inclusion and exclusion criteria, no animal studies could be identified. The protocol including a flow diagram of the search strategy, screening, evaluation, as well as the inclusion and exclusion criteria can be found in Appendix A.

Experimental data was extracted manually and digitally (FIGURE DIGITALIZER V1.0, Hongxue Cai, Mathworks, MA, USA) by independent surveyors. Not all of the studies provided sufficient information (e.g., only statistical values) to extract all parameters for the investigated time intervals.

We introduced a set of quantities to specify the spatio-temporal resolution. The mean tissue velocity ($v_{\mathrm{tisMean}}$) was calculated by dividing the tissue displacement ($∆L_{\mathrm{tis}}$) by the time interval (∆t) in which this displacement occurred during the gait cycle (Equation (1)):

(1) $$v_{\mathrm{tisMean}}=\frac{∆L_{\mathrm{tis}}}{∆t}\;\left[{\mathrm{ms}}^{-1}\right].$$

Equation (2) describes the spatio-temporal resolution of the US sensor system, recording frames at a chosen frame rate (fps). The covered distance (spatio-temporal resolution) per frame (dpf_t_) was calculated by dividing the mean tissue velocity ($v_{\mathrm{tisMean}}$) by the selected frame rate:

(2) $${\mathrm{dpf}}_{t}=\frac{v_{\mathrm{tisMean}}}{\mathrm{fps}}\;\left[m\right].$$

The number of recorded frames during the time interval is given as:

(3) $$\mathrm{fpt}=\frac{∆L_{\mathrm{tis}}}{{\mathrm{dpf}}_{t}}\;\left[-\right].$$

## 3. Results

### 3.1. System Designs and Form Factors

The utilized US systems and their settings are presented in Table 1. Two different US platforms (Echo Blaster by Telemed and ProSound by Hitachi-Aloka) are mainly used for studying muscle and tendon functions during movement. Both systems can be classified as “commercial systems for research purposes” [19], since they have add-on research interfaces. These add I/O functionality for researchers to enable synchronization of US data between different devices (e.g., force plates, 3d motion capture systems, etc.; Figure 3) and authorize access to radio frequency (RF) data for back-end processing.

**Figure 2:**
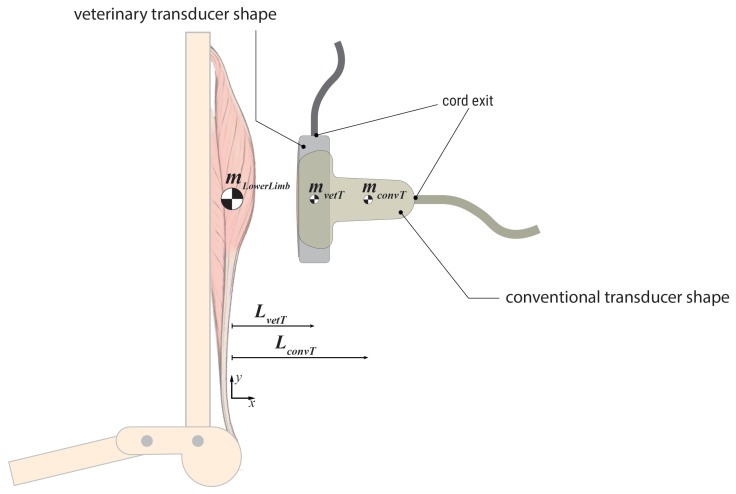
Transducer form factors and their influence on image quality: To build less-interfering setups and reduce imaging bias caused by momentum on the probe head, the lever arm between the centers-of-mass (CM) of moving body parts ($m_{\mathrm{LowerLimb}}$) and ultrasound transducer probes ($m_{\mathrm{vetT}}$–CM veterinary transducer, $m_{\mathrm{convT}}$–CM conventional transducer) should be as small as possible. Veterinary transducers have shorter lever arms ($L_{\mathrm{vetT}}$) than conventional probes ($L_{\mathrm{convT}}$).

Except for Suzuki et al. [20], research groups used single-transducer arrangements to record muscle and tendon functions. They predominantly applied linear array transducers with a size of 40–60 mm and 96–128 imaging channels. The reviewed studies indicate that flat veterinary transducer probe shapes (Figure 2) have advantages over classical linear array transducer shapes [21,22].

As presented in Table 1, most mounting devices are hand-tailored to fit the anatomical form. Elastic straps or compressive self-adhesive bandages are used for probe fixation during motion. For example, Ishikawa et al. [23] specified the weight of 130 g for their probe fixation, including the probe head.

### 3.2. Recording Muscle and Tendon Tissue Dynamics

We present mean measured fascicle velocities and calculated frame-rate-dependent parameters for full stance and stride phases in Table 2, and for critical time intervals during the gait cycle—where maximum tissue velocities occur—in Table 3.

With the exception of Bohm et al. [7], all the studies considered in this review investigated muscle functions in plantarflexors of the lower limbs (Figure 1). First, the MTUs in plantarflexors are main contributors to human locomotion [35,36,37]. Second, muscle fascicle lengths in the soleus (SO), medial gastrocnemius (MG), lateral gastrocnemius (LG) and tibialis posterior (TP) rarely exceed the size of the linear transducer array at any contraction mode. Thus, muscle fascicle length changes can be measured in full over the entire gait cycle. Bohm et al. [7] investigated fascicle behavior in the longer vastus lateralis (VL) using a larger 192-channel linear array probe (100 mm) by accepting lower frame rates of 43 Hz. However, if fascicle length exceeds the covered area, extrapolation methods must be used to estimate fascicle lengths.

Usually, a US transducer tracks either the muscle fascicle or the muscle–tendon junction (MTJ) movement while 3d motion capture registers the position of the related body segment(s). By combining these measurements, the MTU length can then be estimated, for example, by the method proposed by Hawkins et al. [38]. Others transfer the measured data into a virtual environment (e.g., OpenSim [39,40]) where a musculoskeletal model is scaled to the body anthropometry of the test subject [41,42,43,44,45]. Regarding the calculation of MTU lengths for every time increment, the earlier study of Lichtwark et al. [21] used the estimation method proposed by Grieve et al. [46] to calculate MG MTU lengths. However, there is common consensus in favor of the methodology proposed by Fukunaga et al. [47] to estimate serial-elastic-element (SEE) lengths in the reviewed articles.

Across all surveyed studies US images were recorded at an average frame rate of 80 Hz. Nearly half of the studies investigated human locomotion at average speeds of 3 ms${}^{-1}$, and recorded US images at average frame rates of 60 Hz. Studies that investigated higher locomotion speeds recorded images at frame rates up to 100 Hz and more. Particularly, it should be noted that all studies showed spatio-temporal resolutions in the millimeter range.

## 4. Discussion

Muscles contract during locomotion. Thus, the activated muscle shortens and aponeuroses, and tendon(s) change in length. Thereby, the net output of an activated MTU depends on the force–velocity relation [48], the force–length relation [49], the muscle–tendon length [50], the contraction mode (e.g., eccentric, concentric, isometric contraction [51]), and contraction history effects (e.g., force enhancement and depression [52], fatigue [53], as well as tendon hysteresis effects [54]). Hence, the interaction of the whole MTU and all its respective components is responsible for the resulting movement, where the storage and release of elastic energy are also key [3,4,5,55].

Studying these phenomena in vivo during movement requires a wearable US research system with a wide range of view and a high spatio-temporal resolution. Currently available sensor systems are not able to record displacements of muscles, aponeuroses, and tendons simultaneously along the whole MTU, or can only do so to a limited extent. Although imaging system requirements for biomechanics applications are unique, there are several recent developments in US system design which can be leveraged.

### 4.1. System Designs and Form Factors

US imagers as used for biomechanics research (Table 1) such as the Echo Blaster (Telemed) or the ProSound (Hitachi-Aloka) are “commercial systems for research purposes”. They have add-on research interfaces but cannot be reconfigured or do not provide access to transmit operations (TX) and receive operations (RX) due to hardware constraints [19]. Biomechanics researchers are thus left only with the possibilities of finding either more advantageous arrangements for bulky transducers or increasing frame rates by reducing the range of view and image quality. Opening TX operations would allow scientists to better adapt their setups (e.g., by choosing wave forms and steering or focusing the beam at certain areas). Moreover, access to raw RX data would enable the testing of new signal processing methodologies and algorithms. Hence, the development of US imaging platforms for biomechanical research should be driven by the need for more flexibility in parameter settings and access to raw imaging data. Boni et al. [19] defined three key features of open-platform US scanners:

- Customization of transmit waveform (open TX operation);
- Access to pre-beamformed raw data (open RX data-sets); and
- Ability to implement real-time imaging.

**Figure 3:**
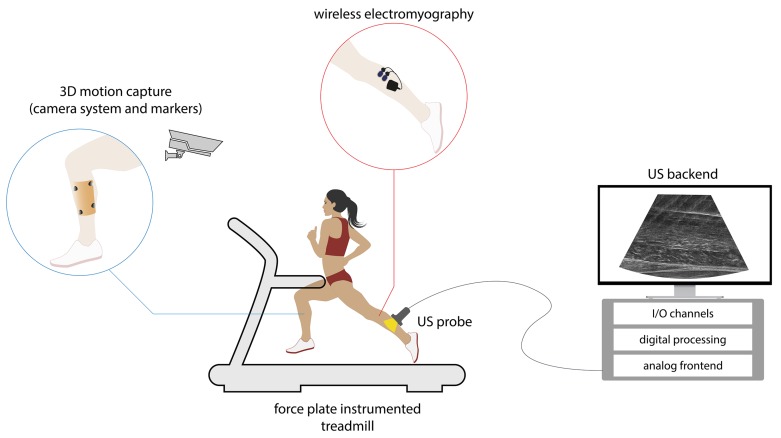
Movement science laboratory using ultrasound measurement during locomotion.

US imagers as applied in movement science work with classical US design concepts. Bulky transducer probes connect to backend systems by analog cable harnesses (Figure 3). These backend systems are powered externally via cable. They mostly have limited internal data storage to save still images for offline analysis and record images at low frame rates. All these characteristics cause obtrusive test setups for the use of US during locomotion. Currently, this impediment is handled by splitting the systems in two. The probe and its connecting cable harnesses are placed as closely as possible to the center-of-mass (CM) of the moving body parts (Figure 2) while the heavier backend system is stored securely outside the testing area. Cronin et al. [31] used a different setup by placing a 5-kg backpack containing the backend system on to the moving subject. However, load carrying affects gait [56]. Such a setup still includes a cable system that interferes with movement: coaxial cable connection between the backend system and the probe; power cable between the backend system and the grid; USB data cable between the backend system and the PC.

Transducer probes of conventional US systems are ergonomically shaped and designed to be hand-operated on patients’ skin for diagnosis. In contrast, the probes of US systems in biomechanical research need to be fixed to the skin during motion (e.g., in order to guarantee reproducible image quality over several stride cycles). Probe mounting is a key task, and must be as unobtrusive as possible to avoid interfering with natural movement patterns while simultaneously ensuring stable fixation to the region of interest. The form factor of veterinary probes (Figure 2) allows the placement of transducers in closer proximity to the body. Cord exits in veterinary probes are in line with the linear array and therefore interfere less with moving body parts. Both characteristics reduce torque on the transducer while in motion, thus decreasing imaging errors.

Movement science needs unobtrusive biomonitoring. This means that sensors are placed in close proximity to bodies and do not interfere with the actions of a subject. In terms of in-vivo biomonitoring human and animal movement, observation devices are still in early stages [14,15,17]. Research demands the development of more parasitic methodologies as defined by Benini et al. [57]. This implies that while US sensors will still be perceptible by test subjects, their size, weight, and structure will not seriously interfere with movement patterns. Their power consumption can range up to a maximum of a few milliwatts with the current energy density in batteries.

### 4.2. Recording Muscle and Tendon Tissue Dynamics

In anatomical images of muscles and tendons during locomotion, the movement of tissue structures (e.g., muscle fascicles) and anatomical landmarks (e.g., MTJs) are recorded. The range of view of US imagers used for biomechanical research allows the observation of tissues within a spatial range between a few millimeters up to approximately 100 mm [58]. A new ultrasound system class for locomotion research in humans should aim to record a range of view of at least 250 mm. This length covers large areas of the MTU in the human plantarflexors [59,60,61] and allows full simultaneous imaging of its components and landmarks. These properties might change for veterinary applications, as MTUs lengths range up to 700 mm and more [11] in large animals.

A US imager for biomechanical research records displacements of muscles and tendons. Hence, frame rates need to be adjusted as tissue velocities rise or spatio-temporal resolution requirements for post-processing change. High-frame-rate imaging in muscle tissues has been exploited dominantly in elastography [62] or studies on cardiac muscles (e.g., pulse wave propagation) in humans [63,64] and animals [65]. Deffieux et al. [66] studied in-vivo muscle contractions of the human biceps brachii at frame rates of 1500 Hz. Their investigations achieved spatio-temporal resolutions in the micrometer range. This setup was needed to dissolve the contraction and relaxation of muscle fibers. In contrast, locomotion research works with conventional US systems (Table 1) which allow spatio-temporal resolutions in the millimeter range, as presented in Table 2 and Table 3. Due to hardware constraints, frame rates are limited. Pushing them beyond 80 Hz with the used system platforms affects image resolutions and decreases the range of view (e.g., because scanlines are reduced).

Lichtwark et al. [21] investigated MG fascicles at locomotion speeds of 2.08 ms${}^{-1}$ while capturing images at frame rates of 25 Hz. Increasing frame rates to 150 Hz for imaging mean states and 250 Hz for imaging critical states would lead to significant improvements in spatio-temporal resolution:

- Mean states recorded at 150 Hz: ${\mathrm{dpf}}_{t}=0.31\times 10^{-3}$ m, fpt=46 (respectively at 25 Hz: ${\mathrm{dpf}}_{t}=1.89\times 10^{-3}$ m, fpt=7);
- Critical states recorded at 250 Hz: ${\mathrm{dpf}}_{t}=0.59\times 10^{-3}$ m, fpt=21 (respectively at 25 Hz: ${\mathrm{dpf}}_{t}=5.96\times 10^{-3}$ m, fpt=2).

In conclusion, research investigating muscle and tendon functions in vivo should aim to record images at frame rates in the kilohertz range. With the special demands of movement science (e.g., wide range of view, unobtrusiveness, mounting, etc.) and current US technologies [67,68], we therefore recommend targeting spatio-temporal resolutions of at least ${\mathrm{dpf}}_{t}=0.5\times 10^{-3}$ m. This corresponds to required frame rates of 150 Hz for locomotion speeds at 3 ms${}^{-1}$. To sufficiently record critical states during the gait cycle or higher locomotion speeds, frame rates of 250 Hz and above might be necessary. Findings for optical systems recording human locomotion using passive markers have shown similar requirements [69].

The ability to capture US images at very high frame rates (100 Hz to 10 kHz) [67,70] is already a feature in today’s high-end research systems [19], and is being implemented in large commercial devices (e.g., Aixplorer by Supersonic Imaging).

### 4.3. Bringing Research Demands into System Form Factors

Measuring the muscle and tendon functions of humans and animals during locomotion poses different technical requirements for US imagers to those for which existing systems have usually been designed. On the one hand, the need for high frame rate, wide range of view, and raw data access would be best met by state-of-the-art, high-channel-count research systems such as Vantage-256 (Verasonics), DiPhAS (Fraunhofer IBMT), SARUS [71], or ULAOP-256 [72]. To the best of our knowledge, there are not yet any handheld mobile systems available on the market that can support such high frame rates.

On the other hand, the need to move without restriction for unhindered measurements requires a free measurement setup. This rules out all the research systems mentioned above, as they consist of large heavy boxes containing the imaging systems’ electronics to which transducers, power supplies, and personal computers (PCs) are connected via cables. By contrast, mobile handheld systems such as Lumify (Philips), MobiUS PE System (MobiSante), or iQ (Butterfly Network) consist of a digital US probe connected to a smartphone. These are light enough to be carried by the subject. However, the digital transducer probe itself is bulky and the transducer opening is ill-placed to attach the probe to the body without interfering with measurements. Moreover, these systems typically do not provide access to raw US data and have processing restrictions (e.g., limited frame rates) due to thermal power constraints [73]. One main issue for the inclusion of high-frame-rate imaging into a system form factor, while meeting the constraints of movement science—and the constraints of portable systems in general—is the huge size of raw image data (>100 MB) and the data rates (>10 GB/s) that sensors produce for processing.

Recent developments in US system design [13] also combine the flexibility of software-defined ultrafast imagers with cost-efficient and miniaturized digital transducers [74]. Current system design trends [19] such as extended numbers of channel systems, hybrid computation approaches (hardware-accelerated vs. software-defined systems), and system design partitioning can contribute to overcoming impediments in biomechanics applications to image whole MTUs at high frame rates.

## 5. Conclusions

This review investigated the current state of applying ultrasound (US) as a research tool to study muscle and tendon functions during locomotion. In terms of biomonitoring muscle and tendon dynamics, science is still in its early stages as US observation devices do not meet the requirements to record tissue structures sufficiently. Biomechanical research demands the development of unobtrusive, wide range of view, and ultrafast US imaging systems. We suggest the consideration of the following recommendations during the development of new US sensor systems for movement science:

- Research studying muscle and tendon functions should aim to record images at frame rates in the kilohertz range.
- Frame rates of at least 150 Hz should be used to reach spatio-temporal resolutions of ${\mathrm{dpf}}_{t}\;=\;0.5\times 10^{-3}$ m. To record tissues at critical states or higher locomotion speeds, frame rates of 250 Hz or more might be necessary to reach the same spatio-temporal resolution.
- The range of view should cover the area of whole muscle and tendon complexes. To record muscle and tendon dynamics sufficiently, we recommend a range of view for US imaging devices of at least 250 mm. This might be substantially larger (700 mm and more) for research on large animals.
- The development of new US imaging solutions in movement science should be driven by the need for more flexibility in parameter settings and access to raw imaging data (open US imaging platforms as defined by Boni et al. [19]).
- The design of a new US system class targeting biomechanical applications must be as unobtrusive as possible in order to avoid interfering with natural movement patterns while simultaneously assuring stable probe fixation to the region of interest.

Hybrid design approaches comprising mobile, ultrafast US hardware and advanced image processing create opportunities to solve previous shortcomings. By following these development guidelines, future US imagers could help to expand the existing knowledge of human and animal movement.

## Figures and Tables

**Table 1:** US sensor system and transducer specifications as well as operating parameters in the biomonitoring of muscle and tendon dynamics during locomotion.

Study	US System	US Transducer	Arrangement	Mounting	Center Frequency (MHz)	Frame Rate (Hz)
*Suzuki 2019* [20]	ProSound α7	60 mm, linear array *(UST-5712)*	double	scratch-build fixture,	7.5	110
	*Hitachi-Aloka (Tokyo, JP)*	50 mm, linear array *(UST-567)*		transmission gel, bandage		
*Lai 2018* [24]	Echo Blaster 128	60 mm, linear array,	single	-	7	80
	*Telemed (Vilnius, LT)*	96 channels				
*Bohm 2018* [7]	MyLab60	100 mm, linear array *(LA923)*	single	neoprene plastic cast,	10	43
	*Esaote (Genova, IT)*	192 channels		elastic straps		
*Swinnen 2018* [25]	Echo Blaster 128 CEXT	60 mm, linear array	single	tape, elastic bandage	8	86
	*Telemed (Vilnius, LT)*	*(LV7.5/60/128Z-2)*				
*Maharaj 2016* [26]	Echo Blaster 128 UAB	96 channels	single	plastic mould, bandage	6	80
	*Telemed (Vilnius, LT)*	*(LV7.5/60/96)*				
*Cronin 2016* [27]	Acuson P300	50 mm	single	elastic bandage	7.5	42
	*Siemens (Erlangen, DE)*					
*Sano 2015a* [28]	ProSound: C3cv/α10	40 mm/60 mm, linear array	single	custom-made Styrofoam cast	13	58/65
	*Hitachi-Aloka (Tokyo, JP)*	20–30 g				
*Lai 2015* [29]	Echo Blaster 128	60 mm, linear array,	single	self-adhesive bandage	7	80
	*Telemed (Vilnius, LT)*	96 channels				
*Sano 2015b* [30]	ProSound α10	60 mm, linear array	single	custom-made support device	13	117
	*Hitachi-Aloka (Tokyo, JP)*	custom-made, 180 g				
*Cronin 2013* [31]	Echo Blaster 128	60 mm, linear array	single	US-system in backpack (5 kg),	7	80
	*Telemed (Vilnius, LT)*	96 channels		compressive bandage		
*Farris 2012* [32]	-	linear array	single	-	8	50
	*Telemed (Vilnius, LT)*	*(LV7.5/60/96Z)*				
*Giannakou 2011* [33]	SSD-4000	42 mm, linear array	single	lightweight foam fixation,	7.5	43
	*Hitachi-Aloka (Tokyo, JP)*			hook-and-loop straps, elastic bandage		
*Cronin 2011* [22]	Echo Blaster 128	60 mm, linear array	single	compressive bandage	7	80
	*Telemed (Vilnius, LT)*	96 channels				
*Lichtwark 2007* [21]	Echo Blaster 128 UAB	60 mm, linear array	single	bandage	7	25
	*Telemed (Vilnius, LT)*	128 channels		*Coban (3M, St. Paul, MN, USA)*		
*Ishikawa 2007a* [23]	SSD-5500m and Prosound α10	60 mm, linear array	single	polystyrene supporting	10	96–196
	*Hitachi-Aloka (Tokyo, JP)*			device (130 g incl. probe-end)		
*Ishikawa 2007b* [34]	SSD-5500	60 mm, linear array	single	-	7.5	96
	*Hitachi-Aloka (Tokyo, JP)*					
*Lichtwark 2006* [8]	Echo Blaster 128 UAB	60 mm, linear array	single	bandage	7	25
	*Telemed (Vilnius, LT)*	128 channels				

**Table 2:** Mean fascicle velocities and mean calculated frame-rate-dependent parameters for full stance or stride phases. Note that not all studies examined provided sufficient information (e.g., only statistical values) to extract all parameters for the investigated time intervals.

Study	No. Subj.	Locomotion Speed	Phase	Fascicle	Δt ${}^{1}$ (s)	ΔL_tis_${}^{2}$ (m)	v_tisMean_ ${}^{3}$ (m/s)	dpf_t_ ${}^{4}$ (m)	fpt ${}^{5}$ (-) (fps (Hz))
*Ishikawa 2007a* [23]	8	6.5 m/s	stance	MG ${}^{7}$	$149\pm 17\times 10^{-3}$	$12.5\times 10^{-3}$	$83.89\times 10^{-3}$	$0.87\times 10^{-3}$	14 (96)
		*TM run ${}^{6}$*						$0.49\times 10^{-3}$	25 (169)
*Suzuki 2019* [20]	7	5 m/s	stance	MG	$160\times 10^{-3}$	$9\times 10^{-3}$	$56.25\times 10^{-3}$	$0.51\times 10^{-3}$	17 (110)
		*TM run, forefoot strike*							
*Swinnen 2018* [25]	19	$3.8\dot{8}$ m/s	stance	MG	-	$16\pm 4.1\times 10^{-3}$	$72.3\pm 20.3\times 10^{-3}$	$0.84\times 10^{-3}$	19 (86)
		*TM run, rearfoot strike*							
*Sano 2015a* [28]	22	3.86 m/s	stance	MG	$205\pm 23\times 10^{-3}$	$3.51\times 10^{-3}$	$17.12\times 10^{-3}$	$0.29\times 10^{-3}$	11 (58)
		*TM run*			$208\pm 10\times 10^{-3}$	$6.64\times 10^{-3}$	$31.92\times 10^{-3}$	$0.55\times 10^{-3}$	12 (65)
*Cronin 2016* [27]	11	$3-3.8\dot{3}$ m/s	stance	SO ${}^{8}$	-	$3\times 10^{-3}$	$29.09\times 10^{-3}$	$0.69\times 10^{-3}$	4 (42)
		*TM run*		MG		$4.63\times 10^{-3}$	$65.45\times 10^{-3}$	$1.55\times 10^{-3}$	2 (42)
*Cronin 2013* [31]	10	$2.8\dot{3}\pm 0.47$ m/s	stance	SO	$254\times 10^{-3}$	$3.25\times 10^{-3}$	$12.78\times 10^{-3}$	$0.16\times 10^{-3}$	20 (80)
		*OG run ${}^{9}$, barefoot*		MG		$12.05\times 10^{-3}$	$47.42\times 10^{-3}$	$0.59\times 10^{-3}$	20 (80)
*Lichtwark 2006* [8]	6	$2.7\dot{7}$ m/s	stance	MG	$288\times 10^{-3}$	$12.84\times 10^{-3}$	$44.60\times 10^{-3}$	$1.78\times 10^{-3}$	7 (25)
		*TM run, incline*							
*Ishikawa 2007b* [34]	7	2.74±0.21 m/s	stance	MG	$296\pm 28.4\times 10^{-3}$	$16\times 10^{-3}$	$54.05\times 10^{-3}$	$0.56\times 10^{-3}$	28 (96)
		*OG run*							
*Lichtwark 2007* [21]	6	2.08 m/s	stance	MG	$312\times 10^{-3}$	$14.73\times 10^{-3}$	$47.20\times 10^{-3}$	$1.89\times 10^{-3}$	7 (25)
		*TM run*							
*Farris 2012* [32]	10	3.25 m/s	stride	MG	-	$13\pm 2\times 10^{-3}$	$28.0\pm 4\times 10^{-3}$	$0.56\times 10^{-3}$	23 (50)
		*TM run*							

${}^{1}$∆t—time interval; ${}^{2}$$∆L_{\mathrm{tis}}$—mean tissue displacement; ${}^{3}$$v_{\mathrm{tissMean}}$—mean tissue velocity; ${}^{4}$${\mathrm{dpf}}_{t}$—mean covered distance per frame; ${}^{5}$fpt—number of recorded frames at selected frame rate; ${}^{6}$ TM run—treadmill run; ${}^{7}$ MG—medial gastrocnemius; ${}^{8}$ SO—soleus; ${}^{9}$ OG run—overground run.

**Table 3:** Mean fascicle velocities and mean calculated frame-rate-dependent parameters for critical time intervals in the gait cycle where maximum tissue velocities occurred. Note that not all studies examined provided sufficient information (e.g., only statistical values) to extract all parameters for the investigated time intervals.

Study	No. Subj.	Locomotion Speed	Phase	Fascicle	Δt ${}^{1}$ (s)	ΔL_tis_${}^{2}$ (m)	v_tisMean_ ${}^{3}$ (m/s)	dpf_t_ ${}^{4}$ (m)	fpt ${}^{5}$ (-) (fps (Hz))
*Lai 2018* [24]	10		stance	SO ${}^{7}$	-	$2.03\times 10^{-3}$	$68.34\times 10^{-3}$	$0.85\times 10^{-3}$	2 (80)
		5 m/s	(ankle moment	MG ${}^{8}$		$2.31\times 10^{-3}$	$75.98\times 10^{-3}$	$0.94\times 10^{-3}$	2 (80)
		*TM run ${}^{6}$*	decline)	LG ${}^{9}$		$2.51\times 10^{-3}$	$56.7\times 10^{-3}$	$0.70\times 10^{-3}$	3 (80)
*Swinnen 2018* [25]	19	$3.8\dot{8}$ m/s	stance	MG	-	-	$218\times 10^{-3}$	$2.53\times 10^{-3}$	- (86)
		*TM run, rearfoot strike*	(0%–30%)						
*Sano 2015a* [28]	22	3.86 m/s	stance	MG	$97\pm 10\times 10^{-3}$	$2.20\times 10^{-3}$	$22.67\times 10^{-3}$	$0.39\times 10^{-3}$	5 (58)
		*TM run*	(push off)		$100\pm 9\times 10^{-3}$	$3.41\times 10^{-3}$	$34.15\times 10^{-3}$	$0.53\times 10^{-3}$	6 (65)
*Bohm 2018* [7]	30	3 m/s	stance	VL ${}^{10}$	$136\pm 18\times 10^{-3}$	$8.5\pm 8.2\times 10^{-3}$	$62.5\times 10^{-3}$	$1.45\times 10^{-3}$	5 (43)
		*TM run*	(active state)						
*Lichtwark 2007* [21]	6	2.08 m/s	swing phase	MG	$88\times 10^{-3}$	$13.12\times 10^{-3}$	$149.14\times 10^{-3}$	$5.96\times 10^{-3}$	2 (25)
		*TM run*	t = 0.6–0.68 s (medial)						
*Maharaj 2016* [26]	15	1.9±0.1 m/s	stance	TP ${}^{11}$	-	$4.5\pm 3.4\times 10^{-3}$	$29.2\pm 6.2\times 10^{-3}$	$0.36\times 10^{-3}$	12 (80)
		*TM walk, barefoot*	(late)						

${}^{1}$∆t—time interval; ${}^{2}$$∆L_{\mathrm{tis}}$—mean tissue displacement; ${}^{3}$$v_{\mathrm{tissMean}}$—mean tissue velocity; ${}^{4}$${\mathrm{dpf}}_{t}$—mean covered distance per frame; ${}^{5}$fpt—number of recorded frames at selected frame rate; ${}^{6}$ TM run—treadmill run; ${}^{7}$ SO—soleus; ${}^{8}$ MG—medial gastrocnemius; ${}^{9}$ LG—lateral gastrocnemius; ${}^{10}$ VL—vastus lateralis; ${}^{11}$ TP—tibialis posterior.

## Abbreviations

The following abbreviations are used in this manuscript:

CM	center-of-mass
LG	lateral gastrocnemius
MG	medial gastrocnemius
MTJ	muscle–tendon junction
OG	overground
RF	radio frequency
RX	receive operation
SEE	serial elastic element
SFDF	superficial digital flexor
SO	soleus
SSC	stretch–shortening cycle
TM	treadmill
TP	tibialis posterior
TS	triceps surae
TX	transmit operation
US	ultrasound
VL	vastus lateralis

## Appendix A

A systematic search (Figure A1) to identify studies that investigated muscles and tendons directly, in vivo, during locomotion using US included the following databases: SCOPUS, Medline, Google Scholar. The database search revealed 172 items, and 4 other sources were added. After title and abstract screening, 29 full-text articles were considered eligible. Twelve were excluded as they did not meet the criteria (locomotion; locomotion speed > 1.9 ms${}^{-1}$; healthy subjects; publication in English). In conclusion, this review covers 17 studies (Table 1).
